# Health, schooling, needs, perspectives and aspirations of HIV infected and affected children in Botswana: a cross-sectional survey

**DOI:** 10.1186/s12887-016-0643-5

**Published:** 2016-07-22

**Authors:** Gabriel Anabwani, Grace Karugaba, Lesego Gabaitiri

**Affiliations:** Botswana-Baylor Children’s Clinical Centre of Excellence, Plot 1836 Hospital Road, Private Bag BR129, Gaborone, Botswana; Baylor College of Medicine, Pediatric Retrovirology, Houston, Texas USA; Baylor International Pediatric AIDS Initiative, Houston, Texas USA; University of Botswana, Gaborone, Botswana

**Keywords:** HIV, Health, School, Needs, Children, Perspectives, Knowledge, Sexuality, Aspirations, Botswana

## Abstract

**Background:**

Antiretroviral treatment means many HIV infected children are surviving with a highly stigmatised condition. There is a paucity of data to inform policies for this growing cohort. Hence we carried out a study on the health, schooling, needs, aspirations, perspectives and knowledge of HIV infected and affected children in Botswana.

**Methods:**

A cross-sectional survey using interviews and focus group discussions among HIV infected children aged 6–8 years versus HIV aged matched HIV uninfected counterparts living in the same households between August 2010 and March 2011. Supplemental clinical data was abstracted from medical records for HIV infected participants.

**Results:**

Nine hundred eighty-four HIV infected and 258 affected children completed the survey. Females predominated in the affected group (63.6 % versus 50.3 %, *P* < 0.001). School attendance was high in both groups (98.9 % versus 97.3 %, *P* = 0.057). HIV infected children were mostly in primary school (grades 3–7) while affected children were mostly in upper primary or secondary grades. Sixty percent HIV infected children reported having missed school at least 1 day in the preceding month. Significantly more infected than affected children reported experiencing problems at school (78 % versus 62.3 %, *P* < 0.001). Twenty-two percent of 15–18 year old HIV infected children were in standard seven and below compared to only 8 % of HIV affected children (*p* = 0.335). School related problems included poor grades, poor health/school attendance, stigma and inadequate scholastic materials. The wish-list for improving the school environment was similar for both groups and included extra learning support; better meals; protection from bullying/teasing; more scholastic materials, extracurricular activities, love and care; structural improvements; improved teacher attendance and teaching approaches. Significantly more HIV infected children reported feeling hungry all the time (50.6 % versus 41 %, *P* = 0.007) and more trouble hearing (26.8 % versus 12.5 %, *P* = 0.028). The mean age for HIV disclosure 10 years was high. Sexual activity (9.2 % versus 3 %, *P* = 0.001) and emotions of anger (71 % versus 55.3 %, *P* < 0.001) were significantly higher among HIV affected children. Future perspectives were equally positive (93 % versus 96 %, *P* = 0.080), were predicated on children’s school performance, self-belief/determination and/or ARVs and preference for medical or military careers was common.

**Conclusions:**

In Botswana almost all school-age HIV infected and affected children are attending school but many face daunting challenges that call for the creation of an empowering, empathetic, supportive, caring, and non-discriminating school environment.

**Electronic supplementary material:**

The online version of this article (doi:10.1186/s12887-016-0643-5) contains supplementary material, which is available to authorized users.

## Background

The successful roll-out of the prevention of mother-to-child transmission and antiretroviral treatment (ART) across Botswana over the last decade and a half has resulted in very few children being infected at birth [1] while those already infected are now surviving into adulthood. HIV infected children may suffer stigma. One study has reported that between 10 and 20 % grade 6 students in nine southern African countries would avoid or shun an HIV positive friend [2]. Disclosure of one’s HIV status can therefore be a big concern for both parents and children. While studies on disclosure of HIV status to children are limited, they suggest that children who know their HIV status have higher self-esteem and that parents who have disclosed the status to their children experience less depression [3]. Further evidence of the benefits of disclosure is provided by a qualitative study involving 16 adolescents living with HIV in Botswana and Tanzania which concluded that disclosure and good HIV-related services provide an important platform for HIV-infected adolescents to resist and cope with HIV stigma [4]. Other research has highlighted the psychological distress experienced by HIV-infected children and youths and suggested that adolescents living with perinatally acquired HIV have higher rates of psychiatric illness than their uninfected peers [5].

Greater understanding of the impact of HIV/AIDS on children is important in the design and evaluation of programmes to support children living in difficult circumstances [6]. Ideally, policies and programs designed to aid this rapidly growing cohort of children should take into account the children’s knowledge, perspective, challenges, and coping mechanisms [7, 8]. To our knowledge, there are no studies in Botswana that have compared the perspectives and knowledge of HIV infected and HIV uninfected children living in the same environment. There is also a paucity of published data comparing the perspectives, knowledge, needs, aspiration and coping mechanisms of HIV infected and affected school children who are living in the same environment.

Thus, the goal of this “Voice of the Child” survey was to help close this knowledge gap by using mixed methods to obtain information directly from a representative sample of HIV infected and affected children of school-going age. The information should inform educational and health policy, provide valuable insights in the needs of HIV infected school going children and form a basis for further research.

### Specific objectives

The specific objectives of the Voice of the Child Survey were to:

1. Investigate the perspectives and experiences of a representative sample of HIV-infected and affected Batswana children and adolescents of school going age (6 to 18 years).
2. Assess the children’s perception of their own general health (physical size, sight, hearing) and dental hygiene
3. Assess the children’s knowledge of HIV and AIDS
4. Assess the felt needs, coping mechanisms and aspirations of HIV infected and affected children.

## Methods

### Target populations, sample size and definitions

The target population consisted a) male and female HIV infected children of school-going age (6–18 years); b) children aged 6–18 years who were not known or suspected to be HIV infected and who were living in the same household as HIV infected children; c) primary caregivers (parents or guardians) of HIV infected children. Eligible HIV infected children had documented HIV positive results on Elisa or PCR testing and had been disclosed to regarding their HIV status. The HIV affected population consisted of children aged 6–18 years who were either known to be free of HIV infection and/or not suspected to be infected and who were living in the same household as a participating HIV infected child and under the care of the same caregiver. If several such children were found in the same household, the one closest in age and/or relationship to the infected child was selected and invited for interview. Primary caregivers were the parents or guardians of the selected HIV infected children. Consent or assent was obtained for all participants. Children were excluded from participation in the study if: age <6 or >18 years; had evidence of severe mental disability; were ill or hospitalised; did not assent to the interview; absence of consent by caregiver. In addition, HIV infected children who did not know their HIV status or without documentation of HIV positive diagnosis were excluded.

According to the data published by the Botswana National HIV treatment programme (*Masa*, December 2007), the largest 5 ART sites in the north of Botswana (Francistown, Mahapye, Serowe, Kasane and Selebi Phikwe) and the largest 5 ART sites in the south (Gaborone, Lobatse, Molepolole, Mochudi and Kanye) accounted for over 90 % of all HIV infected children receiving treatment in the country. Apart from Gaborone and Francistown, all the other ART sites are located in villages of varying size and degree of urbanisation. The largest 10 sites were selected initially for participation because of efficiency and feasibility reasons. In each of these 10 largest ART sites, every second registered patient aged 6–18 was invited to participate until a sample of at least 900 (or roughly 15 % of the registered total in the country) was obtained. For each participating HIV infected child, one qualifying HIV affected child, when available, was invited. Because of unexpectedly slow recruitment in Francistown (due to incomplete patient contact records) the study was extended to include Tutume and Palapye, the next biggest ART sites, both located in large villages. A sample size of 900 HIV infected children and 450 HIV affected children was computed to have sufficient power [9].

### Study design and instruments

This was a cross-sectional psychosocial survey conducted between August 2010 and March 2011 in different sites in Botswana using a range of data collection methods. Interviewer-administered questionnaires were used to collect information from HIV infected and affected children (Additional files 1, 2 and 3). Focus group discussions (FGD) were held for small groups of HIV infected children and HIV affected children. HIV knowledge – covering the three domains of transmission, prevention and anti-retroviral therapy was assessed using a standardized tool. Lastly, a standard form (Additional file 4) was used to abstract medical data (that is, weight, height, disease stage, and latest CD4 count and viral load) from the medical records of HIV infected children. From the data obtained general nutritional status was assessed by calculating weight-for-age and height-for-age z-scores.

Existing validated age appropriate interview tools with closed and open-ended questions were utilized to provide initial format and content for the children’s questionnaires. The sources included the Children’s Self Report and Projective Inventory (CSRPI) [10]; Reaching HIV and AIDS Affected people with Integrated Development and Support (RAPIDS) utilized in Zambia in 2005 [11]; and the Orphans and Vulnerable Children Baseline Survey done in Zambia in 2001 [12]. Demographic data from caregivers was collected to add further context to that of children. These instruments were translated into Setswana (the local national language) and independently back-translated into English to ensure fidelity. Finally, as part of the planned preparation for the field survey, a pilot study involving subjects similar to those targeted for study was used to train the study staff and to fine-tune the final instruments by carefully reviewing them to ensure clarity, consistency and cultural sensitivity. Through this process, it was confirmed that the interviews for eligible children and caregivers would each last between 30 and 45 min.

### Instrument administration process

After obtaining ethical and administrative approvals, an advance visit was conducted to ART sites to further sensitise the local staff about the study, plan for data collection and secure working space for the data collection teams. For each of the study sites, the local facility management appointed a focal point person who facilitated the survey. In all cases, this was a senior nurse or doctor who was well known to the potential respondents and was able to introduce the survey and study staff to them. The study staff worked with the focal point persons to identify the qualifying children, contact their primary caregivers, and invite them to participate in the study. Due to the children’s legal status as minors, access to children was sought through caregivers. Respondents were invited to participate verbally, by letter or telephone contact. For those who could not be contacted that way, a member of the study team accompanied by the local area Family Welfare Educator (FWE), visited their homes and invited them to participate.

When the respondents agreed to participate, written consent was sought from each primary caregiver for the children to be interviewed. Before any interview, interviewers provided detailed information about the study using a standard guide. The subject’s right to refuse to participate and how this wouldn’t disadvantage them or their children was emphasized. Once the interviewers had gained access to the children, the children’s own individual assent to be interviewed was further sought by giving a clear explanation of the research and its purpose and by taking particular care to ensure that children knew they did not have to participate or to answer all the questions.

All interviews were conducted in a quiet room. Assurance was given to both children and their adult caregivers regarding anonymity of the data. Children were interviewed in privacy in the absence of their caregivers in order to obtain their own ideas. Every effort was made to ensure that any emotional stress or disturbance to study participants was minimized. Interviewers were all skilled (trained nurses or social workers) and had counselling and research experience. In the few cases where the respondent being interviewed became emotional, the interviewers would pause for a time, only resuming when the interviewee was calmer and able to make a clear decision to continue. A few respondents who showed signs of emotional distress were counselled and referred for further support as needed. Interview closures were carefully handled. The interviewers would remain available to the respondent for a time after the completion of the interview in case the respondent wanted to ask questions or discuss any issues. A transport reimbursement of P30.00 (US$ 3.0) was paid to each participating caregiver and child.

The process for administration of questionnaires, and extraction of relevant medical data at anti-retroviral treatment (ART) sites was conducted simultaneously by separate teams. Each field team consisted of a supervisor, 4-5 data collectors and a driver. For each team, the supervisor ensured quality control by reviewing all completed questionnaires for omitted or incomplete data prior to transfer for data entry. In addition, site visits by the Principal Investigator (GA) and Study Coordinator (GK) served to identify any deviation from set standards and to provide onsite support to the data collection teams.

Focus group discussions (FGDs) were conducted by an experienced social scientists (GL) using a standardized guide and process. During the quantitative survey, potential participants were invited to participate in FGDs. If they agreed, their contact details were taken; and later – but soon after the quantitative survey - they were contacted for the FGDs. The number of FGDs actually held in each site was determined by the availability of the target populations. Each FGD was planned to consist of 6–10 participants. Children were divided into three separate mixed sex age groups, 6–9, 10–14, and 15–18 years. FGDs were held until saturation was reached. The information obtained was recorded (on tape and by hand written notes) and later transcribed, categorized, analysed and finally triangulated with the information obtained through the other study methods.

### Quality control and assurance

The following quality assurance measures were instituted to ensure that the survey would yield accurate, reliable and valid results:

- The Ministry of Education and Skills Development (MOESD) appointed a Project Review Committee (PRC) in line with the terms of the research contract with the Botswana-Baylor Children’s Clinical Centre of Excellence (COE) which served as a quality control team by monitoring the usage of the approved protocol and by reviewing periodic study reports.
- Data collection tools were developed following extensive reviews of existing psychosocial survey instruments, large scale consultations, and pilot testing to ensure feasibility and stability before the main phase of the survey. The tools were translated into Setswana and back translated into English by a health professional and a linguistic expert with a good understanding of the survey concepts.
- Study staff consisted of experienced clinical research professionals with Good Clinical Practice Certification and qualitative research experts from the University of Botswana. All staff underwent 3-day training on the study protocol and field data collection was preceded by pilot testing which afforded staff with additional training.

### Data management and analysis

Data were recorded on standard study data collection forms, the respective questionnaires serving as both source and clinical record forms or CRFs. At study sites, all forms were reviewed by study supervisors for accuracy and completion. A random sample of completed forms was also reviewed by either the Principal Investigator (GA) or Research Coordinator (GK) with a view to identifying and resolving errors or missing values. Completed data forms were then submitted to the data manager for entry. The forms were date stamped when received by the data entry technician and date stamped a second time when the form has been entered in the database into a tailored Microsoft Access database.

Using SPSS statistical software, programs were created to run logical and missing value error checks on the data files. Edit reports from these programs were used to clean the data with reference to hard copy (source) forms. In addition, periodic quality control analyses were performed on the data to identify and review outliers. Thus the database was updated as necessary. Daily backups of the database were made on two high capacity storage disks and stored separately.

Baseline descriptions were made using percentages, frequencies and means. Quantitative categorical data were compared using Chi-square tests and mean ages were compared using Z-tests. Qualitative data were grouped into mutually exclusive categories according to the central ideas or themes expressed by respondents. These were coded and entered into a computer and later analysed using SPSS software. FGD data were recorded and transcribed. Transcripts were read by the researcher (GL) and coded in the style of grounded theory approach for data analysis. Several category headings were generated from the data and under these all the data were accounted for. An independent researcher (MK) was asked to verify the accuracy of the category system and after discussion minor modifications were made.

## Results

### Study population and setting

Out of 1030 eligible HIV infected children sampled, 999 (97 %) agreed to participate. Of these 15 (1.5 %) were discarded due to the children’s unresponsiveness. Thus analysable data were obtained from 984 HIV infected children, 984 caregivers between 16 August 2010 and 18 February 2011 in 12 ART sites across Botswana (Table 1). Also identified were 258 HIV affected children and all of them agreed to participate. This number was smaller than expected. Recalculating the margin of error yielded a value of 0.056 – which was considered acceptable.

**Table 1:** Participating ART sites and numbers of HIV infected and affected children recruited

Art site	HIV infected (%)	HIV affected (%)
Nyangabgwe Referral Hospital - Francistown	221 (22.5)	71 (27.5)
Botswana-Baylor Children’s Clinic - Gaborone	291 (29.6)	50 (19.4)
Kanye Seventh Day Adventist Hospital	37 (3.8)	5 (1.9)
Athlone Hospital - Lobatse	32 (3.3)	4 (1.9)
Mahalapye District Hospital	64 (6.5)	22 (8.5)
Letsholathebe Memorial Hospital - Maun	62 (6.3)	18 (6.9)
Deborah Retief Memorial Hospital - Mochudi	46 (4.7)	10 (3.9)
Scottish Livingstone Hospital - Molepolole	38 (3.9)	12 (4.6)
Palapye Primary Hospital	52 (5.3)	28 (10.8)
Selibe-Phikwe Government Hospital	49 (5.0)	18 (6.9)
Sekgoma Memorial Hospital (SMH) - Serowe	44 (4.5)	10 (3.9)
Tutume Primary Hospital	48 (4.9)	10 (3.9)
Total	984 (100)	258 (100)

All HIV affected children confirmed living in the same household with the identified HIV infected children; and that their diagnosis had been disclosed fully in 206 (79.8 %) or partially among the remaining children 52 (20.2 %). Majority of HIV affected children were closely related to the HIV infected children: 153 (59.3 %) being siblings, 79 (30.6 %) cousins, 18 (7 %) other relations and only 8 (3.1 %) were unrelated. Majority of affected children 223 (86.4 %) had lived in the same household with HIV infected children for longer than 5 years, 23 (8.9 %) for 6 months up to 2 years, 8 (3.1 %) for 3 to 5 years, 2 (0.8 %) for less than 6 months. The remaining two did not know the length of time they had lived together in the same household.

### Overall demographics

The overall gender distribution and mean age for HIV infected and affected children is shown in Table 2. There were significantly more females among HIV affected children compared to HIV infected children (*p* < 0.001). As expected, majority (91.4 %) of caregivers were females. Most caregivers (71 %) resided in villages, both rural and urban. Their highest educational attainment was as follows: secondary (44 %), primary (39.4 %), none (10.4 %), university 3.4 %, and other (3.8 %). Most caregivers (53 %) were single, 22.1 % were married, 13.1 % co-habiting, 10.2 % widowed, 6 % separated and 1.8 % divorced. Overall, with only 4.4 % in professional cadres, majority of caregivers were either unemployed or engaged in elementary work (17.3 % unemployed; 38.2 % elementary). The mean number of people living per household was 6.48 ± 3.3 (±SD). The number of children aged <18 per household was 3.35 ± 2.29 (±SD).

**Table 2:** Demographic and other variables with outcome variable

Variables	HIV Infected children	HIV Affected children	*p*-value^b^
Sex	N (%)	N (%)	<0.001
Male	489 (49.7)	94 (36.4)	
Female	495 (50.3)	164 (63.6)	
Mean Age (SD)^c^	11.9 (2.8)	13.9 (2.7)	
What do you not like about school?			
Stigma, bullying/teasing	4 (36.4)	-	
Teacher	3 (27.3)	-	
Other children (poor interaction)	3 (27.3)	-	
School is boring	2 (18.2)	1 (50)	
School is hard	1 (9.1)	-	
Takes me away from caregiver	1 (9.1)	-	
Poor school meals	1 (9.1)	-	
Waking up too early for school	1 (9.1)	1 (50)	
Problems faced in school			<0.001
None	214 (22)	97 (37.6)	
Poor Grades	488 (50.0)	71 (27.5)	
Health/Illness/poor attendance	239 (24.5)	12 (4.7)	
Inadequate scholastic materials	104 (10.7)	21 (8.1)	
Negative interaction with other children	65 (6.7)	20 (7.8)	
Lack of friends	63 (6.5)	13 (5.0)	
Stigma	57 (5.8)	7 (2.7)	
Lack of fees	50 (5.1)	13 (5.0)	
Bad behaviour (by other children)	17 (1.7)	11 (4.3)	
Disability/impairments	10 (1.0)	0 (0)	
Bad interaction/relationship with teacher	8 (0.8)	12 (4.7)	
Other	26 (2.6)	9 (3.5)	
Most important things that you feel could be made better for you at school^a^			<0.001
Nothing	188 (19.3)	41 (16.3)	
Extra learning support	176 (18.0)	47 (18.7)	
Improve school meals	144 (14.8)	24 (9.6)	
Protection from bullying/teasing/stigma	127 (13.1)	14 (5.6)	
Adequate scholastic materials/uniforms/free education	117 (12.0)	27 (10.8)	
More leisure, entertainment & extracurricular activities	108 (11.1)	24 (9.6)	
Want teachers to show love/care/patience	102 (10.5)	12 (4.8)	
Make structural and other improvements	101 (10.4)	30 (12.0)	
Improve teachers’ attendance/teaching approaches	58 (6.0)	7 (2.8)	
Keep the school environment clean	52 (5.3)	12 (4.8)	
Provide proper HIV AND AIDS education in school	19 (2.0)	14 (5,6)	
Better relationships with other children at school	24 (2.5)	-	
Other	40 (4.0)	38 (15.1)	
Are you happy living in your home?			0.076
Very Happy	374 (38.0)	118 (45.7)	
Happy	442 (44.9)	101 (39.1)	
Sometimes Happy	107 (10.9)	31 (12.0)	
Unhappy/Sad	51 (5.2)	7 (2.7)	
Very Unhappy	10 (1.0)	1 (0.4)	
Name the most two important things that you feel could be made better for you at home^a^			<0.001
More comfort at home	279 (28.4)	50 (19.4)	
Improve meals at home	214 (21.7)	22 (8.5)	
More leisure, entertainment/play at home	189 (19.2)	20 (7.8)	
Nothing (all is fine)	175 (17.8)	74 (28.7)	
Encouragement/support with school work	147 (14.9)	26 (10.1)	
More space and privacy at home	104 (10.6)	20 (7.8)	
Family to show love/care/patience	92 (9.3)	18 (7.0)	
Adequate scholastic materials/uniforms	66 (6.7)	12 (4.7)	
Harmony in the home/family	23 (2.3)	7 (2.7)	
Medication adherence support	18 (1.8)	11 (4.3)	
Keep home environment clean	17 (1.7)	-	
Other	29 (2.9)	32 (12.4)	
People who are easy to talk to when in case of problems or worry^a^			<0.001
Mother	488 (50)	134 (52.0)	
Aunts	109 (11)	30 (11.6)	
Sisters	59 (6)	38 (14.7)	
Friend	44 (4)	26 (10.0)	
Teacher	36 (3.6)	18 (7.0)	
Cousin	17 (1.7)	16 (6.2)	
Father	42 (4)	16 (6.2)	
Brother	24 (2.4)	14 (5.4)	
Grandmother	76 (7.7)	12 (4.6)	
No one, keep to myself	70 (7.1)	8 (3.1)	
Grandfather	20 (2)	4 (1.6)	
Uncle	10 (1)	3 (1.2)	
Other	24 (2.4)	6 (2.3)	

Note: ^a^Multiple responses allowed. Values in brackets are percentages of frequency of responses divided number respondents. ^b^chi-square *p*-value; ^c^normal *p*-value

### School attendance

**Fig. 1:**
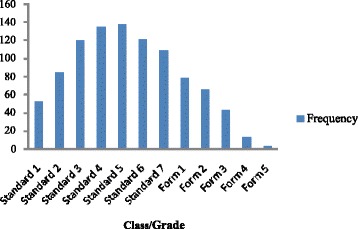
Class distribution of HIV infected children

Of 984 HIV infected and 258 affected children surveyed 974 (98.9 %) and 251 (97.3 %) were currently attending school (*p* = 0.057). Information on school attendance was corroborated by the children’s caregivers. However, the reasons given for not attending school by the few children who were not in school at the time of the survey did not always match those given by their caregivers. For example HIV infected children gave the following reasons for not attending school: failed examinations (3), illness (2), just dropped out (2), pregnancy (1), no funds for school fees (1) and “don’t not know” (1); but their caregivers explanations were: pregnancy (4), not started yet (2), illness (2), financial problems (1), death of parent (1) and child does not like school (1). The 7 HIV affected children who were not currently in school gave the following explanations: failed examinations (5) dropped out of school (1) and did not like school (1). The vast majority of HIV infected (98.9 %) and HIV affected children (97.6 %) were attending a public (government) school (*p* = 0.120). HIV infected children were predominantly in primary school classes (Fig. 1), with standards 3–7 being the most affected while HIV affected children were predominantly in upper primary or secondary school (Table 3). Table 3 also shows that 22 % of 15–18 year old HIV infected children were in standard 7 and below compared to only 8 % of HIV affected children.

**Table 3:** Class distribution by age group

Class distribution	Age of infected children			Total	Age of affected children			Total
	6–9 years.	10–14 years.	15–18 years.		6–9 years.	10–14 years.	15–18 years.	
Special Class	0	0	0	0	0	0	1	1
Standard 1	52	1	0	53	2	0	0	2
Standard 2	74	8	2	84	8	1	0	9
Standard 3	73	47	0	120	4	0	1	5
Standard 4	21	113	1	135	2	11	1	14
Standard 5	1	135	1	137	0	22	0	22
Standard 6	0	106	15	121	0	32	2	34
Standard 7	0	84	25	109	0	25	4	29
Sub-Total	221 (100 %)	494 (90 %)	44 (22 %)	759 (79 %)	16 (100)	91 (80 %)	9 (8 %)	116 (47 %)
Form 1	0	41	38	79	0	19	10	29
Form 2	0	12	54	66	0	4	22	26
Form 3	0	0	43	43	0	0	50	50
Form 4	0	0	13	13	0	0	20	20
Form 5	0	0	4	4	0	0	5	5
Sub-total	0 (0 %)	53 (10 %)	152 (78 %)	205 (21 %)	0 (0 %)	23 (10 %)	107 (92 %)	130 (53 %)
Overall	221	547	198	964	16	114	116	246

To explore the potential impact of HIV on school attendance, HIV infected children were asked to indicate how many days they had missed school during the preceding one month. Excluding those who did not know if they had missed school or not (likely due to young age or forgetfulness), 525 (60 %) of 869 HIV infected children had missed school for at least 1 whole day during the preceding one month – suggesting that absenteeism was a major issue (Table 4). Medical appointments (71 %) and illness (22 %) accounted for the majority of missed school days – suggesting that HIV was the underlying reason for school absenteeism.

**Table 4:** How many days have you missed school during the last 1 month?

Response	Frequency	Percent
None	344	35.8
1–3	451	47.0
4–8	56	5.8
9–12	15	1.6
>13	3	0.3
Don't know	100	10.4
Total	969	100.0

### Perspectives on the school system

When asked if they liked school, almost all of the HIV infected (98.9 %) and HIV affected children (99.2 %) answered in the affirmative (*p* = 0.675). The commonest reason advanced for liking school by HIV infected children was “liked to learn” (64 %). Other reasons included “because of friends” (11 %), “sports” (8 %), “to play with other children” (8 %) and “because of the teacher” (6 %). A smaller number of children mentioned “good food at school”, “school makes me feel better” and “school gives me hope for the future.” Only 11 children said they did not like going to school for reasons that clustered around stigma, bullying, teasing and poor interaction with other children or teachers (Table 2).

When asked if they experienced any problems in school, 22 % HIV infected versus 37.6 % HIV affected children (*p* < 0.001) reported not facing any problems at school. All the remaining children reported facing problems at school (Table 2); and a higher proportion of HIV infected children compared to their HIV affected counterparts reported problems with school grades, health issues or poor school attendance, stigma and disabilities.

To explore if children’s HIV status impacted their children’s relationship with teachers, children were asked two sequential questions: “Do you feel your illness has affected the way your teachers relate to you?” and then, “If yes, how has your illness affected the way your teachers relate to you?” Of 965 children who responded to the question, only 40 (4.1 %) felt their illness had affected the way their teachers related to them. Although 12 of the 40 children were not able to elaborate further when asked the follow-up question the majority (64 %) felt the relationship with their teachers had been affected in a negative or unpleasant way but 36 % reported the opposite - the teachers had changed in a positive way by becoming more supportive to the children.

Both HIV infected and affected children children’s coping strategies in times of stress were largely positive and similar, namely: talking to someone (close relative, class teacher or friend), and working harder at school. However, about 10 % of the children said they just “stayed by themselves.”

Perspectives on the most important things which could be made better at school were explored by asking children to first consider the most important things which they felt could be made better for them at school and then to identify their two top priorities. The results summarised in Table 2 show overall concurrence between HIV infected and affected children’s perspectives. However, proportionately more HIV infected children highlighted school meals, bullying/teasing, more understanding/love by teachers and better teacher attendance and teaching approaches.

#### Home life issues

Participants were asked if they were happy living in their homes. Comparable proportions of HIV infected and HIV affected children reported being very happy, happy, sometimes happy, unhappy or very unhappy (Table 2). Majority of HIV affected children reporting unhappiness attributed this to poor living conditions or ill treatment while the reasons for unhappiness among HIV infected were more varied, including: not well treated at home, poor relationship with other children, inadequate food, no friends or playmates, home disharmony, HIV infection and death of a close relative. When asked how they coped when they felt sad or unhappy at home majority (63.1 %) reported coping strategies that are generally considered as being positive including talking with relatives, crying, playing with friends, writing, drama or music or confronting cause. However, more than one third of the children coped by “doing nothing” or “staying with one self.”

The children’s wish list when they were asked to consider all the things that they felt could be made better for them at home and to name the two most important is summarized in Table 2. Proportionately more HIV infected compared to HIV affected children wished that their homes were improvement to include more comfort, better meals, more leisure/play time, and support with school work. Though small (4.3 % versus 1.8 %), proportionately more HIV affected children wished for more support with medication adherence.

#### General health issues

- Food and Nutrition

In order to assess adequacy of daily food intake, HIV infected children were asked to indicate how many times they ate a meal in any normal day. Over 27 % of HIV infected and 25.7 % of HIV affected respondents reported eating only one or two meals a day (*p* = 0.675). To explore if they ate a balanced diet, we asked what foods the children actually ate in a normal day. The results indicated that only 42.6 % of HIV infected and affected children ate a balanced diet; and only 5 % of the children reported eating fruits and vegetables as part of their diets. When questioned further 13.2 % of the HIV infected and 9.7 % of the HIV affected children said they had slept hungry the previous night (*p* = 0.130). Also 50.6 % of HIV infected children compared to 41.1 % of HIV affected children reported “feeling hungry all the time” (*P* = 0.007

To corroborate the nutrition information we abstracted current weights, heights and ages from the medical records of HIV infected children and used these to calculate weight for age and height for age z-scores. Comparative WHO data are available for children aged 0–10 years (120 months). Therefore we compared data for 208 HIV infected children aged 60–119 months to those of WHO growth standards. The comparisons showed that the mean weight for age of HIV infected children of -1.35 Z-scores was lower than that of the WHO standard of 0.0, indicating wasting among HIV infected children (*p* = 0.0885). Mean height-for-age data for 844 HIV infected children aged 5–18 years was -1.66 Z- scores and significantly lower than that of the WHO standard of 0.0 - confirming significantly more stunting among HIV infected children (*p* = 0.0485).

b) Teeth, Eyes, Ears and Perceived General health

To assess oral health respondents were asked to indicate the times they had brushed their teeth the previous day. The results indicated that of the HIV infected children only 5 and 22.9 % brushed their teeth after breakfast and after dinner, respectively and almost 8 % had not brushed their teeth at all. The corresponding values for HIV affected children were also low at 3.1, 29 and 2.2 %.

For sight issues, respondents were asked if they had any trouble with seeing sometimes. Of the HIV infected children 321 of 984 (26.8 %) reported having trouble seeing sometimes compared to 87 of 258 (33.7 %) HIV affected children. When asked if they had trouble hearing, 264 of 982 (26.8 %) HIV infected versus 32 of 257 (12.5 %) of the HIV affected children (*p* = 0.028). Lastly respondents were asked to indicate what they felt about their general health at the time of interview. Majority of the 957 (93.8 %) HIV infected children said they felt well while 56 of them (5.9 %) said they did not feel well. The corresponding values for 258 respondent HIV affected children were similar at 92.6 and 7.4 %.

### Perceived body size, WHO clinical stage, viral load and CD4 cell counts

Questions on perceived self-body image were asked only to HIV infected participants. Respondents were asked to indicate how they compared their own body size in relation to their classmates and age mates. Of the 979 respondents, about half (52.3 %) indicated that their body size matched the size of their age and class mates; but 36.0 % felt they were smaller while 11.5 % felt they were bigger.

Of the 967 with available clinical data, 58.2 % had early clinical HIV disease (WHO stage 1 or 2) at the time of treatment initiation while the remainder had advanced HIV disease (WHO stage 3 or 4). Nonetheless, at the time of the survey 86.8 % of the HIV infected children had improved their immunological stage to almost normal or near normal levels and 857 of 931(92 %) had undetectable virus (<400 copies/ml).

### HIV knowledge on transmission, prevention and treatment

A standardised tool was used to assess levels of knowledge regarding HIV transmission, prevention and treatment and for each category the results were summarised as poor, moderate or excellent. Knowledge of HIV transmission among 975 HIV infected children was rated as excellent, moderate and poor among 24.5, 32.1 and 43.4 % while the corresponding proportions among 258 HIV affected children were 46.5, 35.2 and 17.8 %, respectively. Knowledge on HIV prevention among 972 HIV infected respondents was rated as excellent, modest or poor among 6.2, 31.5 and 62.3 %, respectively. Among 258 HIV affected respondents the corresponding values were 12, 57 and 31 %, respectively. Knowledge of antiretroviral therapy among 972 HIV infected respondents was rated as excellent in 26.5 %, modest in 44.4 % and poor in 29.1 % respondents. The corresponding rates among 258 HIV affected respondents were 25.2, 47.7 and 27.1 %. Overall, the level of knowledge on transmission, prevention and antiretroviral therapy was surprisingly higher among the HIV affected relative to HIV infected children. Seventy-two percent of HIV infected children stated that they took their HIV medication as prescribed all the time.

### Age at HIV disclosure

HIV infected children were asked when they first learned that they had HIV. If they did not know the exact age, they were probed further to ascertain their ages. If necessary, their medical records were checked for confirmation. This way, the mean age of disclosure for 865 children was determined as being 10.0 ± 2.538 years (Mean ± SD). Over 17 % of the children were disclosed to at ages of 12 years and above.

### Emotions, support and social issues

Perspectives on emotional support were explored by asking questions on current emotional states, support systems and coping mechanisms for HIV infected and affected children. When asked how they felt about their HIV status, over 92 % of 980 respondents described themselves as feeling “fine,” “well” or as having “accepted their status,” while 6 and 1.6 % described themselves as “feeling sad” or “very sad,” respectively. Eight and a half percent of the HIV infected and 8.5 % the affected children reported feeling worried at the time of interview. The worries of HIV infected children revolved around their own and their family’s health while HIV affected children tended to worry more about their HIV infected siblings. When asked if they felt angry in the last one month, 534/966 (55.3 %) HIV infected and 180/253 (71 %) affected children answered in the affirmative (*p* < 0.001). Interestingly, most of the anger of both HIV infected (90.2 %) and affected children (92.2 %) was attributed to family, school and friends (*p* = 0.332). Only 1.7 % of the HIV infected children attributed their feelings of anger to HIV infection or the need to take medication.

Regarding perspectives on the care and support they were receiving at home, 95.5 % of 984 HIV infected children felt they were well cared for and 44 (4.5 %) felt they were not receiving the cared and support they needed. When the later children were probed further on what they thought would help make them feel better at home, 19 of 42 (45.2 %) wished their family would show “more love and care;” 14 of 42 (33.3 %) wished for better meals at home; 5 of 42 (11.9 %) wished their family would “always remind them to take their medication;” and the remaining 4 of 42 (9.5 %) wished for “more safety at home.” Responses from HIV affected children closely mirrored those of HIV infected children. The vast majority (97.6 % of 253) HIV affected children felt that their HIV infected siblings were well cared for at home while a very small number voiced the sentiment that better food, greater medication support, and more love and care would were needed to support their HIV infected siblings in dealing with their condition at home.

Concerning emotional support at school 399 of 974 HIV infected respondents said they could not answer the question as their HIV status was not known at school. Most (92 %) of the remaining 575 said they were well cared for at school while 46 said they were not well care for at school citing the need for “more love care and understanding,” elimination of “bullying and teasing,” “greater learning support,” and better school meals. These perceptions were largely corroborated by HIV affected children: Of the 256 responding affected children, 98 (38.3 %) stated that the HIV status of their HIV infected relatives was not known at school; and of the remaining 158 children 145 (91.8 %) felt their siblings were well care for at school. However 13 of 158 (8 %) affected children stated that that their HIV infected relatives were not receiving good care and wished for elimination of “bullying and teasing;” “educating school personnel on HIV and AIDS issues; and for the school personnel to show “”more love and care.”

Regarding support at community level majority of respondents ((611/980 (62 %) infected and 154/257 (61 %) affected)) stated that the status of the HIV infected children was not known in their respective communities (*p* = 0.769); most of the remaining children (357 infected and 96 affected) stated that they were generally well supported in their communities; while a few (12 infected and seven affected) indicated their wish for less stigma and more community education on HIV issues.

To explore sources of emotional support respondents were asked to identify the people they considered easy to talk in case of need. The results, summarised in (Table 2), show that the preferred sources of support by both HIV infected and affected children are generally identical and include predominantly female relatives followed in decreasing order by friends, teachers and other relatives with fathers, brothers, grandfathers and uncles play relatively insignificant roles. Of concern, some children (mostly HIV infected) felt there was no one they could easily talk to and therefore just kept to themselves.

### Sexual knowledge and activity

We explore sexual knowledge and activity among 576 HIV infected and 174 affected children who were aged 12–18 years at the time of interview. Of these 17 (3 %) HIV infected and 16 (9.2 %) children (*p* = 0.001) said they had ever had sexual intercourse. Sexual intercourse was voluntary among 64 % and 81 % HIV infected and affected respondents, respectively; and only 63 % HIV infected children and 71 % affected children had practiced safer sex by using condoms. One HIV infected and four affected children indicated that they were sexually active in the previous 6 months

### Future perspectives

Over 93 % of HIV infected children felt hopeful about the future despite their illness compared to 96 % HIV affected children (*p* = 0.080). This positive outlook was based on their education/good school performance (39.3 %), self-belief and determination (19.6 %), and on ARVs plus good health (16.9 %). A smaller number indicated they hoped for a cure (4.7 %), that they had been inspired by family or friend (2.5 %), or an HIV positive model (2.4 %), and hope in God (2.3 %). Of the 69 HIV infected children who were not hopeful, the negative future outlook was based largely on poor school grades (52 %) and poor health (23 %). A small number of remaining children did not know the exact cause of their hopelessness; and others were not sure about the future availability of ARVs.

The preferred future careers among 984 HIV infected versus 258 affected children were largely similar: the medical field (39.6 % versus 39.1 %, *p* = 0.884); military (27.6 % versus 15.9 %, *p* < 0.001); academia (13 % versus 17.4 %, *p* = 0.069); business (4.6 % versus 6.2 %, *p* = 0.291). Both HIV infected and affected children looked forward to having families of their own (87.8 % versus 89 %, *p* = 0.597); and children of their own (80 % versus 83.7 %, *p* = 0.180).

### Qualitative findings

A total of 25 focus group discussions were held, 19 for HIV infected children, and 6 for HIV affected children. Qualitative findings corroborated and complement the quantitative findings. Almost all HIV infected and affected children reported liking school for various reasons that included: learning new things, learning to write and read and meeting new friends. They also saw education as an avenue for improving their living conditions. One respondent said, “*I go to school. I like it because I want to have the future. I will be educated and knowing what I want*.” And another: “*I like school because I learn and I socialize with my friends at school.”*

HIV infected children reported no major problems in the school environment. However, some children complained that when they missed school for medical reasons, some teachers coerced them to disclose their status by insisted on seeing their health records. Other children cited being bullied and stigmatised: One said: *“Some of the pupils shout at me at school saying that I have HIV because of sores on my head*.” Some children willingly disclosed their HIV status without parental knowledge: *“I told the class teacher and not friends. I haven’t told the friends because friends are untrustworthy. They may laugh at me when they are with others.”* Some children opined that regular medical check-ups were causing problems for some children. One child stated: *“I only see problems with regular check-ups. I miss the lessons and others will do the subjects and when I come back they will be far.”*

Regarding the most important improvements in schools, sentiments such as the following were expressed “*They must improve the food we eat. The beef we eat is not good. It makes* us *sick*. I want it to be taken out. “And also: *“I also support the one who talks about books. Truly there are no books and the education offered at school is not satisfactory due to lack of books and absenteeism of the teacher due to sickness.”*

Children reported that their general health was mostly fine. However, some of the commonly cited health issues were eye and ear problems, headaches, flu, cough, and skin problems. Most HIV infected children reported taking their medications as prescribed: “*I never miss but sometimes I take my pills late because I come late from playing or I forgot while playing.”* And*: “I take it late and at times I miss.”* Nonetheless, participating in schools trips was an issue for some HIV infected children who did not want to reveal their status by taking ARVs in public: One child said: *“I have never gone on school trips.”*

School teachers were playing an important role in ensuring medication adherence for some children. For example, one child said: *“I take the pills with me. I give them to the teacher and she will tell me when it is time for medication. I tell her that I take my pills at half past 6, and then she sets her alarm.”* Still, contrasting views were expressed in focus groups regarding what role, if any, schools should play to support HIV infected and affected children: One child said: *“Students should be taught (in school) that living with the virus does not mean that one is going to die.”* Another opined: *“I don’t see any way in which the school could help me.”*

Overall, HIV infected and affected children knew various safer sex methods including consistent condom use and monogamy, and abstention. However, information on their own sexual activity was muted in during focus group discussions. Only one child reported ever engaging in sex.

Although most children said they had accepted their HIV positive status, some children had unanswered questions concerning how they had acquired the virus. One child said: *I haven’t yet accepted that I am like this. Maybe as time goes on, I will understand.”* Another posited: *“I have accepted myself. But I ask myself questions. I wonder how I got the virus and I haven’t gotten any answers and this makes me to be moody sometimes.”*

Regarding home support, most HIV infected children said they felt generally cared for in dealing with their illness. However, as the following quotes illustrate, some children felt that they were not adequately supported in dealing with their illness: One child said: “… *but at home I am not satisfied. I don’t get much care particularly from my uncles. They are not interested in my life. They never check my school work to see whether I am passing or what”.* Another said: *“At home my brother is cruel to me. He hides my medications. At school and in the community, it is ok.”* And yet another said*: “At home I can say I get support from mum but dad is not supportive. When I ask him why he doesn’t support me he says he knows what he is doing. I just watch.”*

Although most children were hopeful about their future because they were doing well at school, some said because of their poor school performance, chances of getting a good job were low. The following are quotes of hope: “*I will be working, having a family and there will be peace in the family.”* And: *“I see it as good. I will have a job regardless of whether I have the virus.”* Other children were less hopeful: “*It is difficult. I fail at school.” And: “I have given up. I hear people saying that a child usually resembles his father. Like my father drinks beer and I think I will be a drunkard like my father.”* And*: “The way I see I don’t see any future for myself because of the way I perform at school. If I am not passing well I can’t get a good job.”* Most children wanted to have a family and children. However, some had different views as illustrated by the following quotes: *“I want to be married but I don’t want children.”* And: *“I want to marry and have three children; I can’t manage a huge number of children.”*

Some HIV affected children voiced concerns and worries regarding their HIV infected siblings. One said: *“When she is sick I feel a bit down because she is the person I have a close relationship with, we are the only girls in a family and I wonder that if she dies who is going to be my friend in the family when she is gone.”* Another said: “I feel the pain and hurt and sometimes afraid that he will die.”

Many HIV affected children reported playing active caregiver nurturing roles for their infected siblings: Participation of affected children in the care for infected children. These included reminding the infected children to take medication, making sure they have eaten before they take medication and administering medication when the parents are not present*: “When the schools are closed I give her medication, make sure he has eaten before taking medication and giving him food.”* Also: *“In the absence of my mother I give her medication, I bathe her before she goes to school and I check her homework to see if it’s up to date.”* And: *“I woke up in the morning and warm water for him, then I cook and wake him up to bath and eat then after that I take him out to fresh air and play with him.”*

## Discussion

Literature on the schooling, health, knowledge, aspirations and perspectives of HIV infected and affected children is limited. A number of studies have provided insights on HIV related stigma in schools and the importance of HIV disclosure to both children and their parents [2–5]. To our knowledge this report is the largest and most comprehensive attempt at documenting the felt needs, knowledge, hopes, support systems, emotional wellbeing and coping mechanisms of HIV infected and affected children living in the same environment.

As the target population were school children, we initially considered carrying out a school-based study. However, we soon realised that school-based research would pose major ethical issues regarding, disclosure, privacy and confidentiality. In the end we settled on a methodology that made it possible to access HIV infected children of school-going age efficiently by utilising central records and children’s ART clinics, with the help of experienced HIV care health personnel. Utilisation of a mixed methods approach was chosen to ensure triangulation and to minimise the risk of method specific gaps. This choice was vindicated by the finding in this study that children were more forthcoming in revealing their sexual activity during one-on-one interviews than during focus group discussions.

We believe that the study sample was both robust and representative of HIV infected and affected children in Botswana – a country that is 57 % urbanised [13]. All the 984 HIV infected children were drawn from 12 sites from across Botswana. The two largest study sites (in the cities of Gaborone and Francistown) contributed 52 % of the study population. Many of the patients attending these urban centres are drawn from the surrounding rural and semi-urban areas; and all the ten sites are located in villages of varying size and level of urbanisation. All qualifying HIV affected children living in the same households as the index HIV infected children were recruited.

School attendance rates of 98.9 and 97.3 % among HIV infected and affected children were equally high with almost all attending public schools. This information was obtained directly from the children and it was corroborated by their caregivers. High attendance among the HIV infected is likely due to the fact that they were receiving ART and were generally healthy. Indeed, the medical records showed near normal CD4 cell counts in 86.8 % and undetectable viral loads in 92 % of this population. Most of the children also liked going to school and saw it as an avenue for improving their lives. This high school attendance means that schools provide a unique opportunity to impact the wellbeing of HIV infected and affected children.

The mean age for HIV infected children of 11.9 years was significantly lower than that for HIV affected children of 13.9 years (*p* < 0.001); and HIV affected children were predominantly female (63.6 % versus 50.3 %, *p* < 0.001). This discrepancy may have been caused by selection bias: During focus group discussion, we learned that many HIV affected children, particularly females, were playing caregiver roles. As the average number of children aged <18 years per household was 3, in choosing children closes in age to the index case, we believe caregivers tended to choose older ones with a preference for females as they were already participating in the care of their HIV infected relatives.

School absenteeism due to medical or pharmacy appointments was high among HIV infected children and posed the twin risks of inadvertent disclosure of the children’s HIV status to schools with consequent stigmatisation. Frequent medical appointments were identified by the children themselves as being disruptive to their education process leading to poor grades. The finding that 22 % of 15–18 year old HIV infected children are in standard 7 and below compared to only 8 % of HIV affected children (Table 3) is evidence that HIV infected children are more likely to be held back at school (*p* = 0.335). This could be minimised by scheduling non urgent appointments (laboratory tests and ARV refills) to weekends and school holidays. Scheduling of appointments this way would require close collaboration between caregivers and health personnel as well as supportive policy guidelines by the Ministry of Health. Interestingly, class teacher absenteeism, though not targeted for study, was frequently mentioned by children as constituting an additional impediment to learning.

The results show high levels of hunger particularly among HIV infected children; and anthropometric measurements recorded during the survey confirm high levels of underweight and stunting among HIV infected children compared to WHO standards. Hungry children cannot concentrate on their studies. These findings should not be surprising as these children were mostly living in households with low or unstable income. In the short term, improvement in the quality and quantity of school meals may address the immediate nutritional needs of these children. In the long term, hunger-stricken children need to be identified and assisted with appropriate nutrition education and food support. Policies regarding food support for HIV infected children should consider these findings.

More HIV infected than affected children reported having visual and hearing impairments. Regardless of HIV status, reported tooth brushing habits suggest poor dental hygiene. As good hearing and sight are essential for learning, school-based health screening for these and other impairments should be implemented with urgency. Any identified children with hearing and/or sight impairments should be assisted in receiving the care they need.

The mean age at HIV disclosure of 10 ± 2.538 years is an issue of concern. According to the American Academy of Pediatrics, when disclosure is not done early and properly, involved children can suffer serious emotional and psychological damage [3]. Ideally, the process of disclosure should start much earlier – taking into consideration a child’s cognitive development - and it should be done in such a way that builds on the child’s strength.

In general the levels of knowledge on HIV transmission, prevention and treatment were fairly good. Surprising, HIV affected children had significantly higher knowledge levels even though they do not directly come in contact with the health care system. This difference could be explained by two ways. First HIV affected children were significantly older; and second they played important caregiver roles, likely at the same time, imbibing HIV related knowledge from the caregivers.

Most HIV infected and affected children reported generally being happy and receiving the support they needed at school, home and in their communities. The preferred sources of support in both schools and home are mostly female relatives, friends and teachers. Of concern is the fact that fathers seem to play a very minor role in supporting their children.

Another issue of concern is that many children (mostly HIV infected) felt there was no one they could easily talk to and therefore just “kept to themselves;” others reported feeling sad, anger and having many unanswered questions regarding their HIV status. Teasing and bullying – affecting mostly HIV infected children, seem to be prevalent in schools. Additional issues voiced particularly by HIV infected children included poor school meals, teacher absenteeism, the need for remedial education, more understanding and the need for teachers to be educated in HIV and AIDS issues. These findings suggest that HIV infected and affected school children are experiencing significant social and psychological stress, particularly in their schools.

When they were invited to choose the two most important things they wanted in their homes and schools HIV infected and affected children gave responses that were significantly different as shown in Table 2 (*p* < 0.001). In the home the children wished for more comfort, better meals, more leisure/play time, and more support with school work and with medication adherence. For the school environment the children’s wish list was dominated by the desire for support to improve their school grades, better scholastic materials, more teaching support, better school meals, elimination of bullying and teasing, more understanding/love by teachers and better teacher attendance and teaching approaches. During focus group discussions some children also included the need to educate teachers about HIV and AIDS so that they treat all students/pupils equally irrespective of their HIV status; as well as providing children with vegetables and fruits, increasing the number of meals eaten at school, and providing clothing for the destitute children. None of these wishes are unreasonable.

Although the results of this study can be generalised to HIV infected and affected children living HIV infected households, the same may not be true for children living in HIV free families. A study including HIV uninfected children living in uninfected households would add value to these findings. However, identifying HIV uninfected households is likely to be a challenge.

## Conclusions

Almost all HIV infected and affected children in Botswana who are aged between 6 and 18 years are attending school and recognise the value of schooling. Many of the children face daunting social, psychological and general health challenges. The schooling environment is a uniquely important opportunity to address the needs of this population. These children are calling for the creation of an empowered, empathetic, supportive, caring, and non-discriminating school system in Botswana.

## Abbreviations

AIDS, acquired immune deficiency syndrome; ART, antiretroviral therapy; ARV, antiretroviral drugs; COE, Botswana-Baylor Children’s Clinical Centre of Excellence; CRF, clinical record forms; CSRPI, children’s self report and projective inventory; FGD, focus group discussion; FHI, family health International; FWE, family welfare educator; HIV, human immune deficiency syndrome; MOESD, ministry of education and skills development; NACA, National AIDS Coordinating Agency; PRC, project review committee; RAPIDS, reaching HIV and AIDS affected people with integrated development and support; SCOPE/OVC, strengthening community participation for the empowerment of orphans and vulnerable children; SPSS, statistical package for the social sciences; UNAIDS, Joint United Nations Program on HIV/AIDS; WHO, World Health Organisation

## Additional files

Additional file 1: Questionnaire for HIV infected children. (DOCX 45 kb) Additional file 2: Questionnaire for HIV affected children. (DOCX 39 kb) Additional file 3: HIV knowledge assessment tool. (DOCX 14 kb) Additional file 4: Medical record data abstraction form. (DOCX 19 kb)
