# Erratum to: Health, schooling, needs, perspectives and aspirations of HIV infected and affected children in Botswana: a cross-sectional survey

**DOI:** 10.1186/s12887-016-0676-9

**Published:** 2016-08-19

**Authors:** Gabriel Anabwani, Grace Karugaba, Lesego Gabaitiri

**Affiliations:** 1 Botswana-Baylor Children’s Clinical Centre of Excellence, Plot 1836 Hospital Road, Private Bag BR129, Gaborone, Botswana; 2 Baylor College of Medicine, Pediatric Retrovirology, Houston, TX USA; 3 Baylor International Pediatric AIDS Initiative, Houston, TX USA; 4 University of Botswana, Gaborone, Botswana

## Erratum

Upon publication of this article [1], it was brought to our attention that there was an error in the Methods section of the Abstract: Children’s age range was incorrectly reported as 6–8 years. The correct age range is 6–18 years.
