# Supplementary material for: Health, schooling, needs, perspectives and aspirations of HIV infected and affected children in Botswana: a cross-sectional survey
Source: BMC Pediatr. 2016 Jul 22;16:106. doi: 10.1186/s12887-016-0643-5 (PMC4957906; doi:10.1186/s12887-016-0643-5)
Supplement: Additional file 1: — Questionnaire for HIV infected children. (DOCX 45 kb) [file 12887_2016_643_MOESM1_ESM.docx]

**THE “VOICE” OF THE HIV INFECTED AND AFFECTED SCHOOL AGE CHILDREN IN BOTSWANA: A CROSS-SECTIONAL PSYCHOSOCIAL SURVEY**

**HIV INFECTED CHILD QUESTIONNAIRE**

**Section 1: Demographic Information**

*The questions are simple and intended to make the responded be at ease.*

Study Number: Date of interview: Name of Interviewer:

| **No.** | **Questions** | **Responses** | **Data Code** |
| --- | --- | --- | --- |
| Q200 | What is your birth date?  In what month and year were you born?  *(if not known, ask for age)* | DOB:  Age:  Don’t know | 88 |
| Q201 | Record Sex of Participant | Male  Female | 1  2 |

**Section 2: Education Issues**

***I would like to ask you some questions about your experiences, feelings, and needs regarding school.***

| Q202 | Are you currently in school?  (If yes, Skip to Q206) | Yes  No | 1  2 |
| --- | --- | --- | --- |
| Q203 | Have you ever been in school?  (If Q203 is yes Skip to Q205) | Yes  No | 1  2 |
| Q204 | Why have you never been to school?  (After Answer skip to Q221) | Not started yet  Death of parents/guardian  Financial problems  Illness  Lack of school space  Lack of family support  Don’t like school  Illness of family member  Other: (specify) | 1  2  3  4  5  6  7  8  9 |
| Q205 | Why are you not currently in school?  (After Answer Skip to Q221) | Awaiting results  Death of Parent (s)  Death of Guardian(s)  Drop out  Failed exams  Got a job  Illness  Lack of family support  No funds for school fees  Other: Specify  Don’t Know | 1  2  3  4  5  6  7  8  9  10  88 |
| Q206 | Do you like going to school? | Yes  No | 1  2 |
| Q207 | If No, what do you not like about school?  (*multiple responses possible*) | Teacher  Homework  Other children  Stigma  Away from family, caregiver  Boring  School is hard  No friends  Other: Specify | 1  2  3  4  5  6  7  8  9 |
| Q208 | If Yes, what do you like about school?  (multiple responses possible) | Teacher  Friends  Like to Learn  Play  Out of the home  Sports  Good food at school  Feel better at school  Other: Specify | 1  2  3  4  5  6  7  8  9 |
| Q209 | What kind of school do you go to? | Public (Government)  Private  Other: Specify | 1  2  3 |
| Q210 | What grade (class) are you in?  *(or last grade completed if between classes)* | Standard 1  Standard 2  Standard 3  Standard 4  Standard 5  Standard 6  Standard 7  Form 1  Form 2  Form 3  Form 4  Form 5  College  University | 1  2  3  4  5  6  7  8  9  10  11  12  13  14 |
| Q211 | How many days have you missed school in the last month? (*If 0, skip to Q213)* | 0  1-4  5-8  9-12  Don’t Know | 1  2  3  4  88 |
| Q212 | Why did you miss school?  (multiple responses possible) | HIV Illness  Other Illness  Doctor/Med Appointment  Family issues  Death / Illness in the family  Out of town  Didn’t’ want to go to school  Other: Specify | 1  2  3  4  5  6  7  8 |
| Q213 | Has your illness ever affected your school (class) performance? | Yes  No | 1  2 |
| Q214 | Has your illness caused you to repeat class? If no skip to 216 | Yes  No  Don’t Know | 1  2  88 |
| 215 | If yes, what do you think would have helped you do better? | Don’t know  State: | 88 |
| Q216a | Do you feel your illness has affected the way other children relate to you? | Yes  No | 1  2 |
| Q216b | If yes, how has your illness affected the way other children relate to you? | State: |  |
| Q217a | Do you feel your illness has affected the way your teacher(s) relate to you? If no skip to 219 | Yes  No | 1  2 |
| Q217b | If yes, how has your illness affected the way your teacher(s) relate to you? | State: |  |
| Q218 | How do you cope? | Talk to a friend  Talk with relative  Cry  Writing/drama/music  Play with friends  Play sports  Stayed to self  Nothing  Other: Specify | 1  2  3  4  5  6  7  8  9 |
| Q219 | Who can you talk to in school that you feel can help when you are upset, sad, or frustrated? | Class Teacher  Guidance/Counseling teacher  Social Worker  Other: Specify  None | 1  2  3  4  77 |
| Q220 | Think about all the things that you feel could be made better for you at school. Name the most important two of these. | State: |  |

**Section 3: Home Life**

***Let us now talk a little about your life at home.***

| Q221 | Tell me about your home. Are you happy living there?  *(If Happy or very happy, skip to Q224)* | Very happy  Happy  Sometimes happy  Sad  Very unhappy | 1  2  3  4  5 |
| --- | --- | --- | --- |
| Q222 | If not happy at home, please tell me why? | State: |  |
| Q223 | How do you cope when you are sad or unhappy? | Talk to a friend  Talk with relative  Cry  Writing/drama/music  Play with friends  Play sports  Stay to self  Nothing  Other: Specify | 1  2  3  4  5  6  7  8  9 |
| Q224 | Think about all the things that you feel could be made better for you at home. Name the most important two of these. | 1. State 2. State |  |

**Section 4: General Health, Nutrition**

***Now let us talk a little about your general health***

| Q225a | Tell me how many times you eat in a day? | Fill in #.  Don’t Know | 88 |
| --- | --- | --- | --- |
| Q225b | Tell me what you eat on a normal day | Meat and meat alternatives  Starches (carbohydrates)  Fruits and Vegetables  All the above | 1  2  3  4 |
| Q226a | Do you feel that by the time you went to bed yesterday you had eaten enough? | Yes  No | 1  2 |
| Q226b | Do you feel hungry a lot of the time? | Yes  No | 1  2 |
| Q227 | When did you brush your teeth yesterday?  (Multiple responses possible) | None  Before breakfast  After breakfast  Before dinner  After dinner  Don’t know | 1  2  3  4  5  88 |
| Q228 | Do you have trouble seeing sometimes? | Yes  No | 1  2 |
| Q229 | Do you have any trouble with hearing? | Yes  No  Don’t know | 1  2  88 |
| Q230 | When you compare yourself to your classmates, do you feel you are the same size as them? Tell me how you compare to them. | Same size  Smaller than them  Bigger than them  Don’t know | 1  2  3  88 |
| Q231 | Thinking about your own general health, what do you feel about it right now? | Feel well  Don’t feel well  Other: Specify | 1  2  3 |

**Section 5: HIV Knowledge, Prevention, Treatment**

***Now let us talk a little about HIV***

| Q232 | What do you know about HIV Transmission?  (See attached checklist) | Excellent  Moderate  Poor  Very poor  No response | 1  2  3  4  99 |
| --- | --- | --- | --- |
| Q233 | What do you know about HIV Prevention?  (See attached checklist) | Excellent  Moderate  Poor  Very poor  No response | 1  2  3  4  99 |
| Q234 | What do you know about Antiretroviral Treatment (ARV)?  (See attached checklist) | Excellent  Moderate  Poor  Very poor  No response | 1  2  3  4  99 |
| Q235 | When were you told you have HIV?  (Probe for age as appropriate) | Age  Event |  |
| Q236 | Do you know names of your ARVs?  (confirm by asking names of meds) | Yes  No | 1  2 |
| Q237 | Do you take your medications as prescribed ALL the time?  ( If yes skip to 240) | Yes  No | 1  2 |
| Q238 | If No, why do you sometimes not take your medications? | Make me feel bad ill/nauseated  I Forget  Others forget to give me Others forget to remind me  Tired of taking medicine  Don’t want to take them  Other Specify | 1  2  3  4  5  6  7  8 |
| Q239 | When you don’t take or forget your medicines, what do you do? | Tell Someone  Take them later  Throw away/Hide them  Other:(specify) | 1  2  3  4 |
| Q240 | If you go on a school trip, who gives you your medications? | Self  Teacher  Don’t take them  Never been on school trip | 1  2  3  4 |

**Section 6: Sexual Knowledge, Activity, Future Views**

***Let’s now talk a little about your feelings and activities regarding sex and family***

| ***Questions in this section 6 should only be addressed to children aged 12-18*** | | |  | |  |
| --- | --- | --- | --- | --- | --- |
| Q241 | Have you ever had sexual intercourse?  *(If No, skip to Q248)* | Yes  No |  | 1  2 | |
| Q242 | If yes, was it voluntary? | Yes  No |  | 1  2 | |
| Q243 | Are you currently sexually active? | Yes  No |  | 1  2 | |
| Q244 | Have you been sexually active in the past 6 months? | Yes  No |  | 1  2 | |
| Q245 | Do you practice safer sex all the time? | Yes  No |  | 1  2 | |
| Q246 | If yes, state the method | State : |  |  | |
| Q247 | Does your sexual partner know you are HIV+? | Yes  No |  | 1  2 | |

**SECTION 7: Emotional Support**

***I would like to ask you some questions about emotions and your support system.***

| Q248 | How do you feel now about your condition? | Very Well/Accepted Status  Well  Fine  Sad  Very Sad | 1  2  3  4  5 |
| --- | --- | --- | --- |
| Q249 | Do you feel cared for in dealing with your condition?  At home  In School  In Community | Yes No  Yes No  Yes No | 1 2  1 2  1 2  1 2 |
| Q250 | If No to any of 249 above, what do you think would help you feel more cared for?  a: At home  b: In School  c: In Community | a: State  b: State  c: State |  |
| Q251 | Who do you find easy to talk to when you have a problem or a worry regarding your condition? | Mother  Father  Sister  Brother  Aunt  Uncle  Cousin  Friend  Teacher  Religious authority  Nurse  Doctor  No one, keep to myself  Other: Specify | 1  2  3  4  5  6  7  8  9  10  11  12  13  14 |
| Q252 | Do you prefer to be alone, or playing with other children? | Alone  Play with others  Both | 1  2  3 |
| Q253 | Who do you usually play with? | Mom  Dad  Sister  Brother  Friend  Cousin  Other: Specify | 1  2  3  4  5  6  7 |
| Q254 | Would you say that you feel worried?  (If no, Skip to Q256) | Yes  No | 1  2 |
| Q255 | If Yes, what do you worry about? | Family  Health  Friends  Other : Specify | 1  2  3  4 |
| Q256 | Do you ever feel very angry?  ( If no, Skip to Q258) | Yes  No | 1  2 |
| Q257 | If so, what do you get angry about? | Family  Health  School  Friends  Other: Specify | 1  2  3  4  5 |

**Section 8: Perspectives on the Future**

***Let’s talk a little about how you see your future.***

| Q258 | What do you do for fun?  *(multiple answers possible)* | Football,  Other sports  Games non-physical  Being with friends, playing  Being with family  Dance  Drama  Sing  Reading  Art /drawing/painting  Writing  Crafts, weaving  Go to Church  Other: Specify | 1  2  3  4  5  6  7  8  9  10  11  12  13  14 |
| --- | --- | --- | --- |
| Q259 | Do you feel hopeful about the future?  (If no Skip to 261) | Yes  No | 1  2 |
| Q260 | If so, what makes you feel hopeful? | State: |  |
| Q261 | If not, why are you not hopeful about your future? | State: |  |
| Q262 | Do you look forward to finishing school?  *(question does not apply to children currently not in school)* | Yes  No | 1  2 |
| Q263 | What do you want to be when you grow up or finish school? | Diamond Industry  Business field  Academic field  Military field  Medical field  Others: Specify | 1  2  3  4  8  7 |
| Q264a | Do you look forward to having a family of your own? | Yes  No  Unsure | 1  2  88 |
| Q264b | Do you look forward to having a children of your own? | Yes  No  Unsure | 1  2  88 |
| Q265 | What is something that you are looking forward to doing soon? | State:  Nothing: | 1  2 |
| Q266 | Who do you admire most? | Parent  Family member  Friend  Educator  Coach/Sports figure  Church Related  Celebrity  Medical person  Military person  Other: Specify | 1  2  3  4  5  6  7  8  9  10 |

***Thank you so much for your time and for helping us find out what we can do to help HIV infected children in Botswana.***

Reviewed by: Name and signature Date
