# Supplementary material for: Health, schooling, needs, perspectives and aspirations of HIV infected and affected children in Botswana: a cross-sectional survey
Source: BMC Pediatr. 2016 Jul 22;16:106. doi: 10.1186/s12887-016-0643-5 (PMC4957906; doi:10.1186/s12887-016-0643-5)
Supplement: Additional file 2: — Questionnaire for HIV affected children. (DOCX 39 kb) [file 12887_2016_643_MOESM2_ESM.docx]

**THE “VOICE” OF THE HIV INFECTED AND AFFECTED SCHOOL AGE CHILDREN IN BOTSWANA: A CROSS-SECTIONAL PSYCHOSOCIAL SURVEY**

**HIV AFFECTED CHILD QUESTIONNAIRE**

Section 1: Demographic Information

*I’d like to start by asking a few questions about yourself and your relationship to a child that has HIV infection.*

Study Number: Date of the Interview: Name of Interviewer:

| **No.** | **Questions** | **Responses** |  |
| --- | --- | --- | --- |
| Q300 | What is your birth date?  In what month and year were you born?  *(if not known, ask for age)* | DOB  Age  Don’t know | 88 |
| Q301 | Record Sex of Participant | Male  Female | 1  2 |
| Q302a | Do you live with _____________ (name of the HIV infected child) in the same house hold? | Yes  No | 1  2 |
| Q302b | What do you know about his/her condition?  (if don’t know discontinue interview) | Full disclosure  Partial disclosure  Don’t know | 1  2  3 |
| Q303 | How are you related to him/her? | Sibling  Cousin  Other: Specify | 1  2  3 |
| Q304 | How long have you lived with him/her? | 0 - 6 months  6 months - 2 years  3years - 5 years  Longer than 5 years  Don’t Know | 1  2  3  4  88 |
| Q305 | Do you go to school with children who have HIV? | Yes  No  Maybe  Don’t Know | 1  2  3  88 |

Section 2: Education Issues

*I would like to ask you some questions about your experiences, feelings, and needs regarding school.*

| Q306 | Are you currently in school?  *(If yes, Skip to Q310)* | Yes  No | 1  2 | |  |
| --- | --- | --- | --- | --- | --- |
| Q307 | Have you ever been in school?  *(If 307 is YES, Skip to Q309)* | Yes  No | 1  2 | |  |
| Q308 | Why have you never been to school?  (*After Answer skip to Q310)* | Not started yet  Death of parents/guardian  Financial problems  Illness  Lack of school space  Lack of family support  Don’t like school  Illness of family member  Other: Specify  Don’t Know | 1  2  3  4  5  6  7  8  9  88 | |  |
| Q309 | Why are you not currently in school? | Awaiting results  Death of Parent (s)  Death of Guardian(s)  Drop out  Failed exams  Got a job  Illness  Lack of family support  No funds for school fees  Other: Specify  Don’t Know | 1  2  3  4  5  6  7  8  9  10  88 | |  |
| Q310 | Do you like going to school?  *(If yes, skip to Q312)* | Yes  No | 1  2 | |  |
| Q311 | If No, what do you not like about school?  (skip to 320) | Teacher  Homework  Other children  Stigma  Away from family, caregiver  Boring  School is hard  No friends  Other: Specify | 1  2  3  4  5  6  7  8  9 | |  |
| Q312 | If Yes, what do you like about school?  (If not in school skip to 320) | Teacher  Friends  Like to Learn  Play  Out of the home  Sports  Good food at school  Feel better at school  Other: Specify | 1  2  3  4  5  6  7  8  9 | |  |
| Q313 | What kind of school do you go to? | Public (Government)  Private  Other : Specify | 1  2  3 | |  |
| Q314 | What grade (class) are you in?  *(or last grade completed if between classes)* | Standard 1  Standard 2  Standard 3  Standard 4  Standard 5  Standard 6  Standard 7  Form 1  Form 2  Form 3  Form 4  Form 5  College  University | 1  2  3  4  5  6  7  8  9  10  11  12  13  14 | |  |
| Q315 | Do you think children in your school that have HIV are treated differently? | Yes  No | 1  2 | |  |
| Q316 | If Yes, how are they treated differently? | Ignored  Stigma  Given special attention  Other: Specify  Don’t know | 1  2  3  4  5 | |  |
| Q317a | What problems do you face in school? | Stigma  Attendance  Health/Illness  Grades  Friends  Scholastic materials  Fees  Behavior  Interaction with other children  Other: Specify  None | 1  2  3  4  5  6  7  8  9  10  77 | |  |
| Q317b | How do you cope with those problems you have at school? | Talk to friend  Talk with relative  Cry  Writing/drama/music  Play with friends  Play sports  Stay to self  No Problems  Other: Specify | 1  2  3  4  5  6  7  8  9 | |  |
| Q318 | Who can you talk to in school that you feel can help when you are upset, sad, or frustrated? | Class Teacher  Guidance/Counseling Teacher  Social Worker  Other: Specify  None | 1  2  3  4  5 | |  |
| Q319 | Think about all the things that you feel could be made better for you at school. Name the most important two of these. | 1.State  2.State |  | |  |
|  |  |  | |  | |

**Section 3: Home Life**

***I’m going to ask you some questions about your life and needs at home.***

| Q320 | Tell me about your home. Are you happy living there?  *(If Happy, skip to Q322)* | Very happy  Happy  Sometimes happy  Sad  Very unhappy | 1  2  3  4  5  99 |
| --- | --- | --- | --- |
| Q321 | If sad or not happy at home, please tell me why? | State: |  |
| Q322 | Do you think living with an HIV infected child makes you different from other children? *(if No, skip to Q324)*  *(If* *partial disclosure do not mention* HIV) | Yes  No | 1  2 |
| Q323 | If yes, how does it make you different? | State: |  |
| Q324 | What problems do you face at home because you live with a child who has HIV?  (*If partial disclosure do not mention HIV) (if Response is 5, skip to 326)* | Less attention from caregivers  Stigma  Worry about illness of HIV+ child  Worry about getting HIV  No problems  Other: Specify | 1  2  3  4  5  6 |
| Q325 | How do you cope with these problems in your home? | Go to Adult family  Talk to a friend  Talk to the HIV+ child  Talk to counselor/doctor  Play with friends  Cry  Writing/drama/music  Play sports  Stay to self  Other: Specify | 1  2  3  4  5  6  7  8  9  10 |
| Q326 | Do you think your sibling/cousin/ that is HIV infected is treated differently at home?  *(If partial disclosure do not mention HIV)* | Yes  No  Don’t Know | 1  2  88 |
| Q327 | If so, how is he/she treated differently? | Ignored  Others are scared of them  Treated as if they are sick  Given special attention  Protected more than others  Protected less than others  No different than others  Other: Specify | 1  2  3  4  5  6  7  8 |
| Q328 | Think about all the things that you feel could be made better for you at home. Name the most important two of these. | State: 1  State: 2 |  |

**Section 4: General Health, Nutrition**

***Now let us talk a little about your general health***

| Q329a | Tell me how many times do you eat in a day | Fill in #.  Don’t Know | 88 |
| --- | --- | --- | --- |
| Q329b | Tell me what you eat on a normal day | Meat and meat alternatives  Starches (Carbohydrates)  Fruits and vegetables  All the above | 1  2  3  4 |
| Q330a | Do you feel that by the time you went to bed yesterday you had eaten enough? | Yes  No | 1  2 |
| Q330b | Do you feel hungry a lot of the time? | Yes  No | 1  2 |
| Q331 | When did you brush your teeth yesterday?  (Multiple responses possible.) | None  Before breakfast  After breakfast  Before dinner  After dinner  Don’t know | 1  2  3  4  5  88 |
| Q332 | Do you have trouble seeing sometimes? | Yes  No  Don’t Know | 1  2  88 |
| Q333 | Do you have any trouble with hearing? | Yes  No  Don’t know | 1  2  88 |
| Q334 | Thinking about your own general health, what do you feel about it right now? | Feel well  Don’t feel well  Other: Specify | 1  2  3 |

**Section 5: HIV Knowledge, Prevention, Treatment**

***Let’s talk a bit about HIV.***

| Q334 | Thinking about your own general health, what do you feel about it right now? | Feel well  Don’t feel well  Other : Specify | | 1  2  3 | |
| --- | --- | --- | --- | --- | --- |
| Q335 | What do you know about HIV Transmission?  *(Use attached checklist to grade)* | Excellent  Moderate  Poor | | 1  2  3 | |
| Q336 | What do you know about HIV Prevention?  *(Use attached* checklist to grade*)* | Excellent  Moderate  Poor | | 1  2  3 | |
| Q337 | What do you know about Antiretroviral Treatment?  *(Use attached* checklist to grade*)* | Excellent  Moderate  Poor | | 1  2  3 | |
| Q338 | What do you do to protect yourself at home, knowing your *(relationship to index child)* has HIV infection?  *(If partial disclosure do not mention HIV)* | Wash hands  Don’t share objects  Don’t eat off same plate  Stay away from sick child  Other: Specify | | 1  2  3  4  5 | |
| Q339 | Do you feel the way you relate to *(relationship to index child)* is affected by him/her having HIV infection?  *(If partial disclosure do not mention HIV)* | Yes  No | | 1  2 | |
| Q340 | If yes, why? | State: | |  | |
| Q341 | If No, why? | State: | |  | |
| Q342 | How do you feel when your *(relationship to index child)* is sick? | Sad  Fine  Scared/Worried/Anxious  No Different  Other: Specify | | 1  2  3  4  5 | |
| Q343 | Do you participate in the care for the HIV Infected child in your home when he/she is sick?  *(If partial disclosure do not mention HIV)* | Yes  No | | 1  2 | |
| Q344 | If no, why not? | Scared to get infected  Others care for him/her  Don’t know what to do  Other: Specify | | 1  2  3  4 | |
| Q345 | Do you participate, remind, or give ARV medications to the HIV infected child in your home?  *(If partial disclosure do not mention HIV or ARV)* | Yes  No | | 1  2 | |
|  |  |  |  | |  |

**Section 6: Sexual Knowledge, Activity, Future Views**

*Let’s now talk a little about your feelings and activities regarding sex and family*

| ***Questions in this section should only be addressed to children aged 12-18*** | | | |
| --- | --- | --- | --- |
| Q346a | Have you ever had sexual intercourse?  *(If No, skip to Q351)* | Yes  No  Don’t know | 1  2  88 |
| Q346b | If yes, was it voluntary? | Yes  No | 1  2 |
| Q347 | Are you sexually active now? | Yes  No | 1  2 |
| Q348 | Have you been sexually active in the past 6 months? | Yes  No | 1  2 |
| Q349a | Do you practice safer sex all the time? | Yes  No  Sometimes | 1  2  77 |
| Q349b | If yes, state the method | State: |  |
| Q350a | Does your sexual partner know your HIV status? | Yes  No | 1  2 |
| Q350b | Do you know your sexual partner’s HIV status? | Yes  No | 1  2 |
| Q351a | Do you look forward to having a family of your own? | Yes  No  Unsure | 1  2  88 |
| Q351b | Do you look forward to having children of your own? | Yes  No  Unsure | 1  2  88 |

SECTION 7: Emotional Support Issues

*I would like to ask you some questions about emotions and your support system.*

| Q352a | Do you feel your sibling/cousin is cared for in dealing with his/her condition?  1. At home  2. In School  3. In Community | Yes No Not known  Yes No Not known  Yes No Not known | 1 2 3  1 2 3  1 2 3 |
| --- | --- | --- | --- |
| Q352b | What do you think would help him/her feel more cared for?  1. At home  2. In School  3. In Community | 1. State  2. State  3. State |  |
| Q353 | Who do you find easy to talk to when you have a problem or a worry?  (*multiple responses possible)* | Mother  Father  Sisters  Brother  Aunts  Uncle  Cousin  Friend  Teacher  Religious authority  Nurse  Doctor  No one, keep to myself  Other: Specify | 1  2  3  4  5  6  7  8  9  10  11  12  13  14 |
| Q354 | Would you say that you feel worried?  (If no, Skip to Q357) | Yes  No | 1  2 |
| Q355 | If Yes, what do you worry about? | Family  Own Health  HIV+ Child’s Health  Friends  Other: Specify | 1  2  3  4  5 |
| Q356 | Do you ever feel very angry?  ( If no, Skip to Q359) | Yes  No  No Response | 1  2  99 |
| Q357 | If so, what do you feel angry about? | Family  Health  School  Friends  Other: Specify | 1  2  3  4  5 |

Section 8: Perspectives on the Future

*Let’s talk a little about how you see your future.*

| Q358 | What do you do for fun?  *(Multiple responses possible)* | Football, sports, physical  Games non-physical  Being with friends, playing  Being with family  Dance  Drama  Sing  Reading  Art /drawing/painting  Writing  Crafts, weaving, basketry  Church  Other: Specify | 1  2  3  4  5  6  7  8  9  10  11  12  13 |
| --- | --- | --- | --- |
| Q359 | Do you feel hopeful about your future? | Yes  No  Don’t Know | 1  2  88 |
| Q360 | If, so what makes you feel hopeful? | State:  Nothing  Don’t Know | 1  2  88 |
| Q361 | If not, why are you not hopeful about your future? | State:  State:  Don’t Know | 1  2  88 |
| Q362 | Do you look forward to finishing school?  (Question not relevant to children not currently in school) | Yes  No  Don’t Know | 1  2  88 |
| Q363 | What do you want to be when you grow up or finish school? | Diamond Industry  Business field  Academic field  Military field  Medical field  Other: Specify  Don’t know | 1  2  3  4  8  7  88 |
| Q364 | What is something that you are looking forward to doing soon? | State:  Nothing | 1  2 |
| Q365 | Who do you admire most? | Parent  Family member  Friend  Educator  Coach/Sports figure  Church Related  Celebrity  Medical person  Military person  Other: State | 1  2  3  4  5  6  7  8  9  10 |

*Thank you so much for your time and for helping us find out what we can do to help HIV infected and affected children in Botswana.*

Reviewed by: Name and signature Date
