# Supplementary material for: Health, schooling, needs, perspectives and aspirations of HIV infected and affected children in Botswana: a cross-sectional survey
Source: BMC Pediatr. 2016 Jul 22;16:106. doi: 10.1186/s12887-016-0643-5 (PMC4957906; doi:10.1186/s12887-016-0643-5)
Supplement: Additional file 3: — HIV knowledge assessment tool. (DOCX 14 kb) [file 12887_2016_643_MOESM3_ESM.docx]

**HIV KNOWLEDGE ASSESSMENT TOOL**

**TRANSMISSION:**

1. Unprotected sexual intercourse with an infected partner
2. Mother to child transmission
3. Contact with infected blood e.g. blood transfusions, through cuts

*Key*: Mentions all = Excellent

Mentions 2 = Moderate

Mentions 1 = Poor

**HIV PREVENTION:**

1. Condom use
2. PMTCT
3. Abstinence
4. Avoiding contact with infected blood
5. Male circumcision
6. Behaviour change e.g. reducing MCP

*Key*: Mentions all = Excellent

Mentions 3= Moderate

Mentions 2 or 1= Poor

**ART**

1. Drugs used to reduce the amount of HIV in the body (makes the virus weak/go to sleep)
2. Lifelong treatment
3. Drugs taken same time every day
4. Drugs which are not supposed to be missed

*Key*: Mentions all= Excellent

Mentions 3= Moderate

Mentions 1 or 2= Poor
