# Supplementary material for: Health, schooling, needs, perspectives and aspirations of HIV infected and affected children in Botswana: a cross-sectional survey
Source: BMC Pediatr. 2016 Jul 22;16:106. doi: 10.1186/s12887-016-0643-5 (PMC4957906; doi:10.1186/s12887-016-0643-5)
Supplement: Additional file 4: — Medical record data abstraction form. (DOCX 19 kb) [file 12887_2016_643_MOESM4_ESM.docx]

**THE “VOICE” OF THE HIV INFECTED AND AFFECTED SCHOOL AGE CHILDREN IN BOTSWANA: A CROSS-SECTIONAL PSYCHOSOCIAL SURVEY**

**MEDICAL RECORD ABSTRACTION FORM (HIV infected children)**

***Information should be extracted from the children’s most recent medical records at study sites:***

DATE: STUDY NUMBER: NAME OF INTERVIEWER:

| **TYPE OF INFORMATION** |  |
| --- | --- |
| SEX |  |
| DATE OF BIRTH |  |
| HEIGHT (cm) |  |
| WEIGHT (kg) |  |
| DISEASE STAGE: **IMMUNOLOGICAL** |  |
| DISEASE STAGE: **CLINICAL** |  |
